# Do eye trackers estimate eyeball rotation? The relationship between tracked eye image feature and estimated saccadic waveform

**DOI:** 10.3758/s13428-025-02862-5

**Published:** 2025-10-30

**Authors:** Marcus Nyström, Diederick C. Niehorster, Roy S. Hessels, Richard Andersson, Marta K. Skrok, Robert Konklewski, Patrycjusz Stremplewski, Maciej Nowakowski, Jakub Lipiński, Szymon Tamborski, Anna Szkulmowska, Maciej Szkulmowski, Ignace T. C. Hooge

**Affiliations:** 1 Lund University Humanities Lab, Box 201, 22100 Lund, Sweden; 2 Department of Psychology, Lund University, Box 201, 22100 Lund, Sweden; 3 Experimental Psychology, Helmholtz Institute, Utrecht University, Heidelberglaan 1, 3584 CS Utrecht, The Netherlands; 4 Institute of Physics, Faculty of Physics, Astronomy and Informatics, Nicolaus Copernicus University in Toruń, ul. Grudziądzka 5, 87-100 Toruń, Poland; 5 Inoko Vision, ul. Mickiewicza 7/17, 87-100 Toruń, Poland; 6 Tobii AB, Box 743, 182 17, Danderyd, Sweden

**Keywords:** Retina, Eyeball, Eye tracking, Rigid, Saccade waveform

## Abstract

The eyeball is not rigid and deforms during saccades. As a consequence, the saccade waveform recorded by an eye tracker may depend on which structure of the eye is used to estimate eyeball rotation. Here, we systematically describe and compare signals co-recorded from the retina, the cornea (corneal reflection, CR), the pupil, and the lens (fourth Purkinje reflection, P4) during saccades. We found that several commonly used parameters for saccade characterization differ systematically across the signals. For instance, saccades in the retinal signal had earlier onsets compared to saccades in the pupil and the P4 signals. The retinal signal had the smallest saccade amplitude and reached the peak saccade velocity earlier compared to the other signals. At the end of saccades, the retinal signal came to a stop faster than the other signals. We discuss possible explanations that may account for the relationship between the retinal signal and the other signals.

## Introduction

What is the relationship between the physical rotation of the eyeball and the signal an eye tracker outputs? This question is critical to address when studying, for instance, saccade dynamics (Tabernero & Artal, 2014; Nyström, C., & Holmqvist, 2013) or for the classification of fixations and saccades (Nyström & Holmqvist, 2010; Larsson, Nyström, & Stridh, 2013). As will be shown in this paper, the answer to the question is also tightly linked to the eye-tracking principle used.

Many eye trackers estimate the orientation of the eyeball or the direction of gaze by mapping features from an image taken of a participant’s eye to gaze locations in the world (for details, *cf.*, e.g., Stampe, 1993; Hansen & Ji, 2010; Holmqvist et al., 2011). Image features can be described as characteristics of an image, e.g., shapes, corners, or regions in an image that share specific properties, and are typically detected and described by computer vision algorithms (Hassaballah, Abdelmgeid, & Alshazly, 2016). In eye tracking, features can refer to local regions in an eye image that correspond to physical parts of the eye, for instance, the pupil (i.e., a hole in the eye enclosed by the inner border of the iris, Merchant, Morrissette, & Porterfield, 1974), the iris (Sigut & Sidha, 2010), as well as reflections off different structures in the eye resulting from external illumination, for instance the reflection off the front of the cornea (known as the corneal reflection, CR, or the first Purkinje image, P1, Merchant et al., 1974; Nyström, Niehorster, Andersson, Hessels, & Hooge, 2022) or the reflection off the back of the lens (the fourth Purkinje reflection, P4, Cornsweet & Crane, 1973; Crane & Steele, 1985). As such, the appearance of an eye-tracking signal depends on, besides the physical eye movement itself, at least the quality of the eye image, the algorithms used to extract the image feature, the movement of the image feature with respect to the eyeball, and the quality of the function used to map image features to gaze locations.

Using features such as the pupil or CR to estimate eye orientation directly and in isolation typically requires a stabilized head, since any head movement relative to the camera makes it difficult to distinguish between eye translation relative to the camera and eye rotation relative to the head (Young & Sheena, 1975). Even a small head translation of 0.1 mm can lead to a 1 deg apparent gaze shift in the eye-tracker signal (Crane & Steele, 1985). This problem can be significantly reduced by using two different features in the eye image that are displaced with the same amount from head translation, but different amounts with eye rotations, thereby allowing the separation of eye translation from eye rotation. Examples of such eye-tracking techniques include the pupil and corneal reflection (P–CR) technique that uses the vector between the centers of the pupil and the corneal reflection (Merchant, 1969; Merchant et al., 1974) and the dual Purkinje (DPI) technique (Crane & Steele, 1985), which employs the vector between the first (CR) and the fourth Purkinje image (P4).

To be able to reflect gaze direction and eyeball rotation during saccades accurately, the above approaches for eye tracking rely on the rigid eye assumption (Nyström, Hooge, & Andersson, 2016b; Hooge, Holmqvist, & Nyström, 2016, where the eyeball is considered a rigid body and all parts of the eye undergo the same rotation during a saccade. However, several papers have invalidated this assumption. Co-recording monkeys with scleral search coils and the EyeLink 1000 P–CR eye tracker, Kimmel, Mammo, and Newsome (2012) found that the peak velocity of saccades and the amplitude of post-saccadic oscillations (PSOs) were higher in data recorded with the EyeLink, using the P–CR principle, compared to data recorded with coils, which more accurately estimate the rotation of the whole eyeball. They hypothesized that the high angular acceleration exerted on the globe at the start of saccades would make the pupil lag behind the whole eyeball and lead to a spring-like mechanism accounting for the higher peak velocity in saccades recorded with the EyeLink. Similarly, at saccade offset, when the eyeball abruptly stops, the pupil would continue to move back-and-forth before coming to a full stop, generating higher amplitude PSOs in the EyeLink compared to the coil signal. By direct comparison of the pupil and iris center in eye images from human observers, Nyström et al. (2013) confirmed that the pupil indeed moves relative to the iris center, where the latter was used as a proxy for eyeball rotation.

Comparing the position of the centers of the pupil, the CR, and the P4 in eye images during a saccade, Artal (2014) and Nyström, Hansen, Andersson, and Hooge (2016a) found the appearance of the position-signals to be different in several aspects. First, at the onset of a saccade, the CR started to move first, followed by the pupil, and then the P4. Second, the peak saccade velocity and acceleration estimated from the P4 signal were the highest, followed by the pupil and CR signals. Finally, the P4-signal had longer PSO duration and larger PSO amplitude compared to PSO in the pupil, and in particular, the CR signal. Analogous with the spring-like mechanism for the pupil described by Kimmel et al. (2012) for P–CR eye trackers, the origin behind the differences between CR and P4 signals has been attributed to the non-rigid attachment of the lens to the eyeball (Crane & Steele, 1985; Deubel & Bridgeman, 1995). Importantly, since computing a gaze signal using the P–CR or the DPI principle involves subtracting two signals that differ in both space and time, signals from these eye trackers do not accurately represent the physical motion of the eyeball, but lead to significant overestimation of saccadic parameters such as peak velocity and amplitude. Consequently, both the P–CR and DPI eye tracking principles have been deemed unsuitable to study saccade dynamics (Deubel & Bridgeman, 1995; Hooge et al., 2016).

So far, we have considered eye trackers that image the front of the eye to estimate eye orientation or gaze direction. However, another type of eye tracker images the fundus of the eye, which includes the retina, macula, optic disc, fovea, and blood vessels in the back of the eye (Young & Sheena, 1975). A common implementation of retinal eye tracking is the scanning laser ophthalmoscope (SLO), which estimates eye rotation based on how far the retinal image has translated between consecutively recorded SLO images (Sheehy et al., 2012). Although this technique typically has a low sampling frequency, there are implementations of SLOs with higher sampling frequencies that allow detailed recordings of saccades (e.g., Bartuzel et al., 2020). For instance, Stetter, Sendtner, and Timberlake (1996) used an SLO with a sampling frequency of 1000 Hz, and found saccades recorded with their system to be comparable with saccades recorded by other researchers with a scleral search coil system in terms of amplitude and peak velocity.

In general, however, little is known about how signals recorded from the retina are related to signals recorded from other structures of the eye, such as the pupil, the cornea, and the lens.

For the first time, we will co-record signals from the front (anterior part) and the back (posterior part) of the eyeball to investigate how the signals routinely used for eye tracking relate to each other during saccades. Unlike consecutive recordings with different devices, simultaneous co-recordings allow direct comparison, at the individual saccade level, of how different structures in the eye move relative to each other. Specifically for our study, anterior signals comprise the CR, pupil, and P4, and the posterior signal is a retinal signal recorded with a newly introduced retinal eye tracker with high spatial and temporal resolution (the FreezEye tracker, Bartuzel et al., 2020; Tamborski et al., 2022). The main goal of this paper is to systematically describe properties of the retinal signal during saccades, in relation to signals from the CR, P4, and pupil. Specifically, we will visualize signals from repeated saccades, report commonly used saccade metrics such as saccade amplitude and peak velocity, as well as when saccade onsets and peak velocities occur in the different signals. Moreover, we quantify the dynamics of the signals at the end of saccades. Finally, we discuss possible explanations that may account for the relationship between the retinal signal and the other signals.

## Methods

### Participants, stimuli, and apparatus

Three of the authors, two with long (20+ years) experience from eye tracking, both as researchers and subjects, and one less experienced, took part in the experiment.

Eye movements from the left eye were recorded at 610 Hz with the FreezEye tracker (Bartuzel et al., 2020; Meina et al., 2021, FET), a high-speed retinal eye tracker that does not require participant calibration. The FET outputs eye rotation in degrees. We will refer to the output from the FET eye tracker as the retinal signal.

Simultaneously with the FreezEye recording, the eye was filmed by a CMOS camera (Basler acA800-510um, 1220Hz @ 300x288 pix) through the imaging lens of the retinal eye tracker. The anterior structures of the eye were illuminated by an infrared diode ($\lambda = 810$ nm) positioned adjacent to the imaging lens, serving dual purposes: (1) scattered light from the eye structures provided sufficient contrast for visualizing the iris and pupil, (2) specular reflections from the cornea and lens generated Purkinje reflections. The scattered and reflected light, collected by the imaging lens, was directed to the camera via two dichroic mirrors. At the first dichroic mirror, the retinal signal was transmitted to the scanners and detection system, while the pupil light was reflected to the second dichroic mirror. At the second mirror, the pupil light was transmitted through an additional lens, forming a sharp image of the eye’s anterior structures, including the Purkinje reflections, on the camera detector. The camera focus was individually adjusted for each subject to ensure a clear image of the pupil edge.

**Fig. 1:**
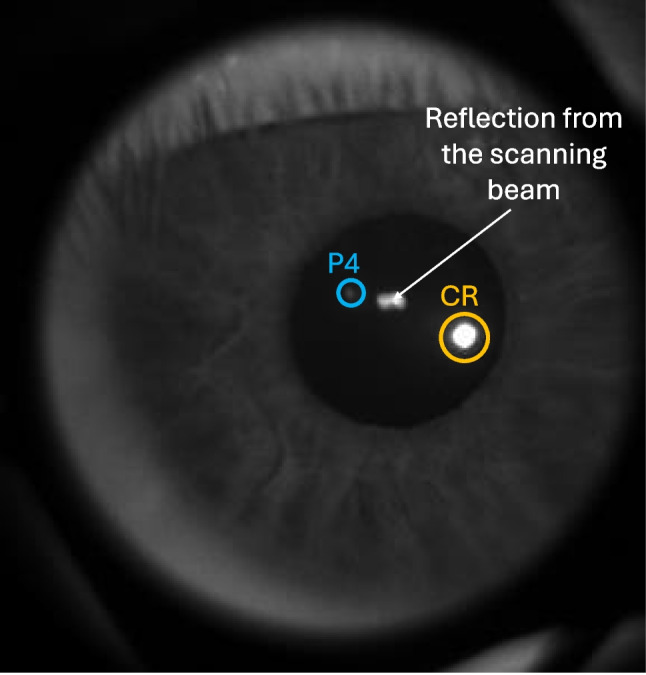
Eye image recorded from the external camera. The centers of the pupil, the corneal reflection (CR, large white dot at the bottom of the pupil marked with an *orange circle*), and the fourth Purkinje reflection (P4, small white dot in the upper part of the pupil marked with a *blue circle*) were computed for each frame. The *white arrow* points to a reflection from the scanning beam of the retinal eye tracker

One frame from the recorded video is shown in Fig. 1, where the aperture of the lens is visible as a dark circle encompassing the eye’s image. For each frame, the center positions of the pupil, CR, and P4 were computed. The camera was synchronized with the retinal tracker by using the same electronic signal to both drive the vertical axis of FET’s MEMS scanners and trigger frame acquisition for the camera.

Stimuli consisted of two fixation markers separated by 2 degrees such that their center of gravity was horizontally and vertically aligned with the center of the image display. The decision to use 2-degree horizontal saccades is motivated as follows: First, in data from P–CR video-based eye trackers, small saccades have been associated with proportionally larger post-saccadic oscillations (PSOs) compared to larger saccades  (Hooge, Nyström, Cornelissen, & Holmqvist, 2015). Moreover, the PSOs are larger in horizontal than vertical saccades. Consequently, potential differences in waveform between the retinal and other signals will likely be more easily observed for small, horizontal saccades. Second, we wanted to present stimuli well within the estimated tracking range of the FreezEye tracker, which is about 8 degrees in the horizontal and vertical direction. Second, we wanted to present stimuli well within the estimated tracking range of the FreezEye tracker, which is about ±4 degrees in the horizontal and vertical direction.

The image display consisted of an LCD display (Sharp LS029B3SX02, 1440$\times$1440 pixels, 2.9", 90 Hz) providing a $16^{\circ } \times 16^{\circ }$ field-of-view. The fixation marker was a red cross with an angular size of 0.75$^\circ$ presented on a dark green background.

### Procedure

Before starting a recording, the operator used a tool to calibrate the retinal eye tracker (synchronize the MEMS scanners with the galvo scanners and obtain the mapping functions from driving signal to optical angles, for details, see Bartuzel et al., 2020; Meina et al., 2021). The calibration procedure was performed once after the eye tracker was turned on.

Participants placed their heads in a chin rest and forehead rest in front of the eye tracker. The operator adjusted the eye tracker’s position on the *x*, *y*, and *z* axes so that the pivotal point of the scanning beam was located in the center of the pupil. Each measurement with the retinal tracker, as with typical ophthalmic retinal imaging devices, was performed with the lights off to maximize the diameter of the participant’s pupil.

During the recording, the participants were asked to perform saccades between the fixation markers at their own pace for 60 s, three times each with a small break in between. As such, 180 s of data were recorded for each participant.

**Fig. 2:**
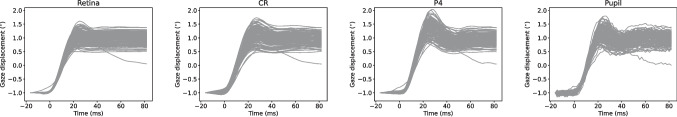
Saccades in the rightward direction for participant S1 as they appear in the retinal, CR, P4, and pupil signals. Each line represents one saccade. Time zero (0) corresponds to the saccade onset in the retinal signal. The saccades in the CR, P4, and pupil signals have been scaled to have the same displacement (i.e., the Euclidean distance from $t_0$ to $t_1$) as their corresponding saccades in the retinal signal, where $t_0=-16.4$ ms and $t_1=80$ ms

### Data processing

The center locations of the CR, pupil, and P4 were computed for each participant and video frame in the external camera, thus providing three signals in units of camera pixels.

Pupil detection occurred in two stages. First, a pupil mask was created by locating the darkest point in the image’s central region, applying flood fill segmentation with a set tolerance, removing small areas, and using a convex hull to smooth the largest contour. Second, contours were extracted from the mask, and the largest contour was approximated as a polygon, and a robust ellipse was fitted to points on this polygon with the RANSAC algorithm (Fischler & Bolles, 1981). For later frames, the ellipse was refined by sampling points on its perimeter, computing intensity gradients along lines perpendicular to the ellipse, and using parabolic interpolation for subpixel accuracy before fitting an updated ellipse (Halır & Flusser, 1998).

For CR detection, the algorithm masked the image outside an enlarged pupil region, identified the pixel with the highest intensity, and computed the radial symmetry center (Parthasarathy, 2012) in a small region around this pixel location, with subpixel localization accuracy.

Depending on whether or not the CR was successfully detected, different approaches were used to detect the P4. If no CR was detected, the algorithm localized the brightest pixel in the full pupil region, whereas if a CR was detected, the brightest pixel within a 20-degree trapezoidal zone opposite the CR from the pupil center was localized. As for CR localization, radial symmetry was then computed to determine the final P4 position. A smoothing process clustered P4 locations over time, selecting the cluster with the least velocity change for better consistency.

After localizing the centers of the pupil, CR, and P4, the algorithm applied post-processing steps to remove outliers by identifying large gradient changes and discarding short, unstable spans. In all the frames, reflections from the FET scanning beam were masked out, exploiting the fact that its intensity varied between the even and odd camera frames.

Saccade onsets and offsets were computed from the retinal signal using the EK-algorithm (Engbert & Kliegl, 2003) with parameters $\lambda =20$ and a minimum saccade duration of 4.92 ms (3 samples at 610 Hz). To prevent PSOs from being detected as separate saccades, a minimum inter-saccade duration of 19.7 ms (12 samples) was used. Only saccades in the retinal signal with a Euclidean distance between the onset and offset between ($[1.4, 2.6]^{\circ }$), performed in the left or right direction (± 20$^{\circ }$), and starting within $<0.3^{\circ }$ from a target location were considered. Finally, we rejected a saccade if any of the signals contained more than one one-sample spike, meaning the distance between two consecutive gaze samples was larger than 2 degrees. After applying these criteria, 200 (out of 231) saccades remained for participant S1, 264 (out of 276) saccades for S2, and 162 (out of 186) saccades for S3.

To decrease the variability of where in the gaze signal a saccade onset was detected, onsets generated from the EK algorithm were refined by considering saccades in the retinal signal to begin when the horizontal gaze velocity exceeded 25$^{\circ }/s$. Gaze velocities were computed by differentiating gaze positions by a third-order Savitzky–Golay filter (Savitzky & Golay, 1964) with a window length of 18.0 ms (11 samples, see Nyström & Holmqvist, 2010). The same filter was used for all signals.

Since only small eye rotations are considered, it can be assumed that there is a linear relationship between eye rotations in the retinal signal and displacement of the CR, pupil, and P4 in the eye image. Therefore, the CR, pupil, and P4 signals were converted from pixels to degrees for individual saccades by applying a scaling factor derived from the retinal signal. The signals were scaled such that the displacement (Euclidean distance from saccade onset to saccade offset) between $t_0$ and $t_1$ was identical for each saccade across all four signals, where $t_0=-16.4$ ms and $t_1=80$ ms, and $t=0$ ms represents the saccade onset. Figure 2 shows saccades in the retinal, CR, P4, and pupil signals, scaled to degrees and selected to be included for further analysis according to the criteria described above.

Finally, we computed the amplitude, peak velocity, and the time of the peak velocity for the selected and scaled saccades in Fig. 2, for all participants. Here, only the horizontal component of the signals was considered. Amplitude was computed as the difference between the smallest and largest gaze displacement values for a saccade, peak velocity as the maximum velocity value during the saccade, and the time of peak velocity as the time where the peak velocity was reached. Note that according to this operationalization of amplitude, two saccades can have similar displacements but different amplitudes, for instance, if one of the saccades has a larger PSO.

Image processing was performed with Python 3.11 and OpenCV (v. 4.11.0.86). Remaining data processing was done with Python 3.12.3.

**Fig. 3:**
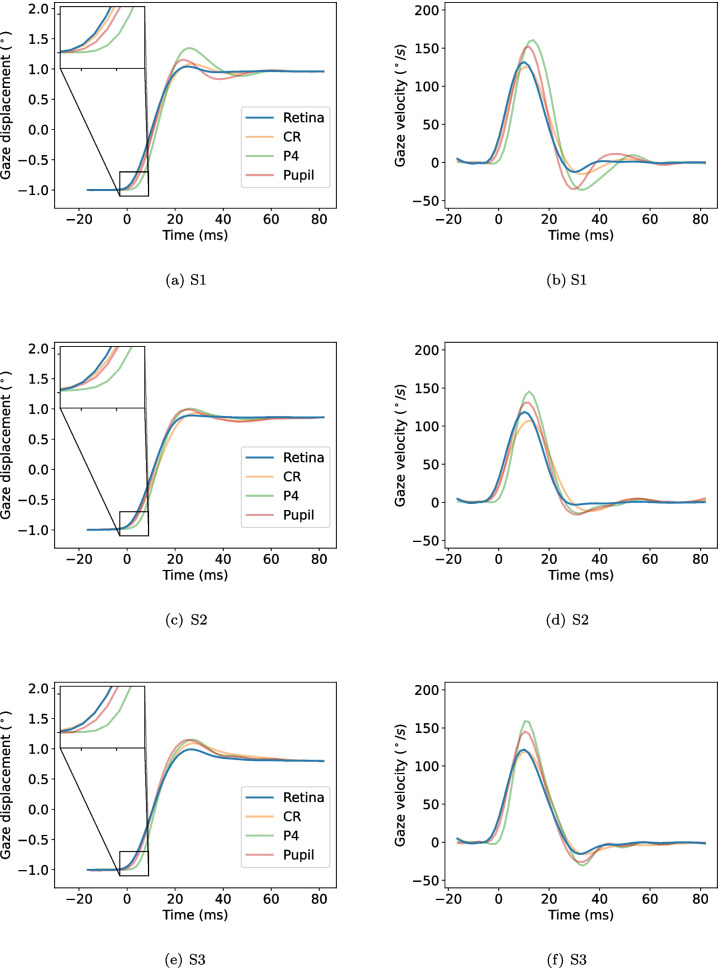
Average saccade waveforms for gaze displacement (*left column*) and gaze velocity (*right column*) from three participants (S1 [first row], S2 [second row], and S3 [third row]) performing self-paced saccades in the rightward direction between two dots separated horizontally by two degrees. Signals from the retina were recorded with the FreezEye tracker (FET), and CR, pupil, and P4 signals were recorded with an external camera. The insets show zoomed-in portions of the signals at the time of the saccade onset. Time 0 corresponds to saccade onset in the retinal signal

## Results

Figure 3 shows average waveforms for saccades from all participants in the rightward direction, for gaze position (left column) and gaze velocity (right column), and for the retinal, CR, P4, and pupil signals. As can be seen from the zoomed-in parts of the plots, saccades in the retinal and CR signals seem to start first, and saccades in the P4 signals seem to start the latest. From the gaze-velocity plots, it is clear that saccades in the P4 signals have the highest peak velocity and saccades in the CR signal have the lowest. At the end of saccades, the retinal signals seem to reach a stable value earlier than the other signals.

**Fig. 4:**
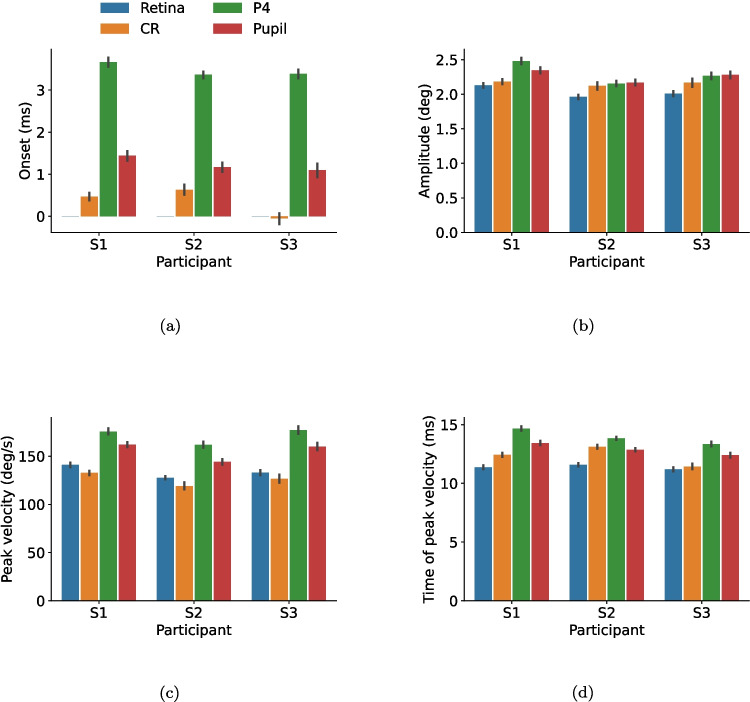
Selected properties of saccades from the retinal, CR, P4, and pupil signals for the three participants (S1, S2, and S3). *Error bars* represent 95% confidence intervals around the mean. Saccade onset in the retinal signal was used as a reference value in (a), and has therefore the value 0 for all participants. Data from both left-, and rightward saccades are included

To quantify and extend these observations, Fig. 4 provides summary statistics for saccades in the different signals, including the onset, amplitude, peak velocity, and the time the peak velocity of the saccade is reached. Here, data from both saccade directions were combined. For amplitude, saccades in the retinal signal had the lowest amplitudes, and saccades in the pupil and P4 signals had the highest amplitudes. Moreover, the velocity peak of saccades in the P4 signal was reached later than in the other signals, where the earliest peak was found for saccades in the retinal signal.

There are also between-participant differences. For example, for S2, the shapes of the pupil and P4 signals seem to match quite well (Fig. 3 (c)). However, this is not the case for S1. In general, however, relative differences across signals and within participants appeared quite systematic (apparent from the similar shape of the bar graphs across participants in Fig. 4).

To quantify the frequency and the damping of the last part of the saccade waveform—the post-saccadic oscillations (PSOs, Nyström et al., 2013; Hooge et al., 2015; Larsson et al., 2013)—we extracted part of each signal from the saccade peak velocity and onward, for each saccade and signal type, and attempted to model it as a damped harmonic oscillator (He, Donnelly, Stevenson, & Glasser, 2010; Tabernero & Artal, 2014 according to

1 $$\begin{aligned} g(t) = A e^{-\gamma t} cos(2 \pi f t - \varphi ) + \beta \end{aligned}$$

where *A* is the amplitude, $\gamma$ is the damping factor, *f* is the frequency, $\varphi$ is the phase, and $\beta$ is a factor accounting for shifts in the signal. However, some of the saccade signals turned out to be poor fits to Eq. 1, which made us abandon this approach. As an alternative method to approximate the damping factor, we computed the time from the peak saccadic velocity to the last sample with an absolute velocity value larger than $10^{\circ }/$s, i.e., when the PSO was considered to end and a more stable period follows. To prevent individual spurious samples from exceeding this value, the average signals were used. Saccades in the retinal signal ended on average earlier compared to saccades in the other signals (CR: 6.7 ms, P4: 5.6 ms, pupil: 8.1 ms, where positive values mean later than the retinal signal).

## Discussion

We asked how signals acquired from a retinal eye tracker were related to signals co-recorded from the cornea (corneal reflection, CR), pupil (pupil center), and lens (fourth Purkinje reflection, P4) during saccades, using a recently developed retinal eye tracker (Bartuzel et al., 2020) together with a high-speed camera filming the front of the eye.

Importantly, we could replicate the findings of Artal (2014) and Nyström et al. (2016a) regarding the relation between the CR, pupil, and P4 signals during saccades. This suggests that the external camera setup and image processing pipeline we have developed provide output in line with previous work and that our setup in the future has the potential to be used as a traditional eye tracker using both the P–CR and the DPI (CR–P4) principles. It should be noted that eye trackers using such differential signals will have saccade profiles more deviant from eyeball rotation compared to the signals presented here; since there are time lags between the CR, P4, and pupil signals (the P4 signal starts to move later than the CR signal at saccade onsets, for instance), such differential signal will significantly overestimate amplitudes and peak velocities (Kimmel et al., 2012; Hooge et al., 2016).

Overall, saccades in the retinal signal started earlier and reached their peak velocity quicker compared to saccades in the other signals. Moreover, saccades in the retinal signal had larger saccade peak velocity than those in the CR signal but smaller than in the pupil and P4 signals. Oscillations after the saccade (PSOs) in the retinal signals came to a stop earlier compared to the other signals.

There are at least two explanations that may be helpful to consider when interpreting these findings. According to the first explanation, a displacement of the retinal image observed by the eye tracker is a good estimate of the physical displacement of the fundus. Since the retina is closely attached to the sclera and together form a larger structure of the eye, one may hypothesize that a displacement of the retina is commensurate with eyeball rotation. As such, we would expect a high agreement between the retinal signal and the CR signal, which has been shown to be the least ‘wobbly’ of the CR, pupil, and P4 signals during saccades (Artal, 2014; Nyström et al., 2016a), and may therefore more closely approximate eyeball rotation. Clearly, our data show little support for this explanation, since there were noticeable deviations between saccade waveforms in the retinal and the CR signals for all participants, both spatially and temporally (cf. Fig. 3). This contrasts the empirical findings by Stetter et al. (1996), who found the amplitude and peak velocity of saccades from a retinal eye tracker to be comparable with those recorded with scleral search coils, which are considered to estimate the orientation of the whole eyeball. Note, however, that unlike the simultaneous recordings we conduct in this paper, Stetter et al. (1996) compared the retinal signal to coil data recorded by other researchers using different participants. Thus, it is conceivable that the similarities (Stetter et al., 1996) report may be specific to the circumstances during which the participants were recorded. It can furthermore be discussed whether signals from the CR and scleral search coils are equally good representations of eyeball rotation, since coils are placed directly on the sclera while the CR reflects off the cornea, which has an elasticity of its own.

The second explanation takes into account that the retina is imaged through the crystalline lens, which translates and tilts during saccades (He et al., 2010; Tabernero & Artal, 2014; Boszczyk, Dębowy, Jóźwik, Dahaghin, & Siedlecki, 2023). According to this explanation, the retinal image observed by the eye tracker is influenced by lens motion on top of whole eyeball rotation. Consequently, one could hypothesize that the waveform of the retinal signal would align the most with the P4-signal, which represents the reflection off the posterior surface of the lens.

This explanation gains some support from simulations by Artal (2014), which predict significant retinal image motion as the optical consequence of lens wobbling. However, our findings showed that saccades in the retinal signal started and ended earlier, and had lower saccade peak velocity compared to the P4 signal.

Recent work has suggested that the P4 is formed by a complex combination of lens translation and tilt (Boszczyk et al., 2023), and the relation between these and the appearance of the retinal image during saccades is not fully understood. Given the relatively higher peak velocity of saccades in the retinal signals compared to the CR signals in our work, it can be hypothesized that part of the lens translation and tilt that contributes to the formation of the P4 also affects the appearance of the retinal signal. This raises the question of how accurately retinal eye trackers represent saccade dynamics (*cf.* Deubel & Bridgeman, 1995; Hooge et al., 2016).

There are additional explanations besides the two introduced above that may account for the findings we obtained. One such explanation is that lens movements do not influence the appearance of the retinal signal, but the retina deforms in its own right during saccades. Indeed, there is evidence that the retina is elastic and deforms when exposed to a force, at least when detached from the rest of the eye (Jones, Warner, & Stevens, 1992). Future studies should probe these potential explanations.

It should be noted that the FET eye tracker used to record retinal signals is designed to be robust against small head rotations (Tamborski et al., 2022). However, the CR, P4, and pupil signals acquired with the external camera are not, as they reflect a combination of head and eye rotations. This is evident in Fig. 2, particularly in the first 20 ms of the signals, where the dispersion of the CR, P4, and pupil signals is higher compared to the retinal signal. This dispersion is likely introduced by small head rotations, despite participants’ heads being stabilized with a chin and forehead rest. Importantly, these head rotations are small and slow compared to saccades and likely have a minimal influence on the overall signal waveform.

In this article, we asked participants to perform horizontal saccades with short amplitudes (2 deg). Future studies could examine the relationship between signals across saccade amplitudes and directions. Such data could clarify whether the relative difference observed in this article would be stable or more pronounced for certain saccade amplitudes/directions. It should also be noted that other signals than the retinal, pupil, CR, and P4 signals may be relevant to consider; perhaps most notably, the iris center signal (Sigut & Sidha, 2010). Since the outer iris border (between the iris and the sclera) is likely more rigidly coupled to the eyeball than the inner iris border (the pupil), the corneal bulge, and the lens, it would be interesting to include the iris center signal in future studies.

### Conclusion

Signals recorded from the retina, cornea (CR), lens (P4), and pupil differ in terms of metrics commonly used to describe saccades. Consequently, researchers should be cautious when interpreting and comparing saccades recorded by eye trackers using different features to estimate eye movements.

## Declarations

### Open Practices Statement

Due to data protection rules, the data cannot be made available, but documented code for analyses in this article is available from https://osf.io/esj58/ and is illustrated with a separate recording made for illustrative purposes. The experiment was not preregistered.
